# Social dominance predicts hippocampal glucocorticoid receptor recruitment and resilience to prenatal adversity

**DOI:** 10.1038/s41598-018-27988-9

**Published:** 2018-06-25

**Authors:** Moshe Gross, Hava Romi, Ayala Miller, Albert Pinhasov

**Affiliations:** Department of Molecular Biology, Ariel University, Ariel, 4070000 Israel

## Abstract

The developing fetus is highly sensitive to prenatal stress, which may alter Hypothalamic-Pituitary-Adrenal (HPA) axis programming and increase the risk of behavioral disorders. There is high variability among the human population, wherein many offspring of stressed pregnancies display resilience to adversity, while the remainder displays vulnerability. In order to identify biological substrates mediating between resilience or vulnerability to prenatal adversity, we exposed stress-resistant Dominant (Dom) and stress-sensitive Submissive (Sub) mice to mild prenatal restraint stress (PRS, 45 min on gestational days (GD) 15, 16 and 17). We hypothesized that PRS would differentially alter prenatal programming of limbic regions regulating the HPA axis and affect among Dom and Sub offspring. Indeed, PRS increased Sub offspring’s serum corticosterone, and exaggerated their anxiety- and depressive-like behavior, while Dom offspring remained resilient to the hormonal and behavioral consequences of PRS. Moreover, PRS exposure markedly facilitated glucocorticoid receptor (GR) recruitment to the hippocampus among Dom mice in response to restraint stress, which may be responsible for their resilience to stressful challenge. These findings suggest proclivity to adaptive or maladaptive prenatal programming of hippocampal GR recruitment to be inheritable and predictable by social dominance or submissiveness.

## Introduction

The developing fetus is highly sensitive to changes in the intra-uterine environment, which may carry life-long consequences for the offspring. While increased basal glucocorticoid levels during pregnancy are considered beneficial to proper CNS development^1^, exposure of the pregnant dam to prenatal stress leads to elevated levels of glucocorticoids^2^, which pass through the placenta to the fetus, influencing prenatal programming of the Hypothalamus Pituitary Adrenal (HPA) axis, responsible in large part for the organism’s style of coping with adversity. Specifically, maternal glucocorticoids have been shown to alter fetal development of the hippocampus^3^, wherein glucocorticoid receptor (GR) activation is considered essential to successful adaptation to stress, by both dampening the initial behavioral stress reaction and by promoting recovery from stress^4–6^. Resultant changes in hippocampal responsiveness to glucocorticoids may alter the offspring’s ability to adapt to stress-inducing stimuli in adulthood, such that stress may trigger the development of behavioral disorders among sensitive individuals.

However, the adverse transgenerational effects of prenatal stress are not uniformly detected in the clinic^7^. While prenatal maternal stress has been shown to cause intrauterine growth restriction (IUGR)^8,9^, long considered to increase the risk of a range of psychiatric^10–12^, metabolic^13–15^ and cardiovascular^16–18^ illnesses among adult offspring, prenatal stress is a weak predictor of adult mental health status^19^. Although prenatal stress is an independent risk factor for psychiatric illness among adult offspring^19^, there is high variability in the stress response among the human population: whereas many offspring of stressed pregnancies display resilience^20^, the remainder may later develop affective disorders^21^. The weak associations between psychopathology and known stress mediators (e.g., glucocorticoids) suggest the induction of a meta-plastic state by prenatal stress among developing offspring that heightens their sensitivity to environmental stressors later in life^19^. Thus, the variable response to prenatal stress and subsequent stressful challenge later in life are likely to be mediated by genetic variation^22^, such that networks of gene-environment interactions may underlie the highly unpredictable effects of a pregnancy challenged by stress^23^. Indeed, the vast genetic diversity of participants in clinical studies of prenatal stress has been identified as a central factor obscuring the pathways by which prenatal stress may engender the development of behavioral disorders in adulthood^19^.

**Figure 1:**
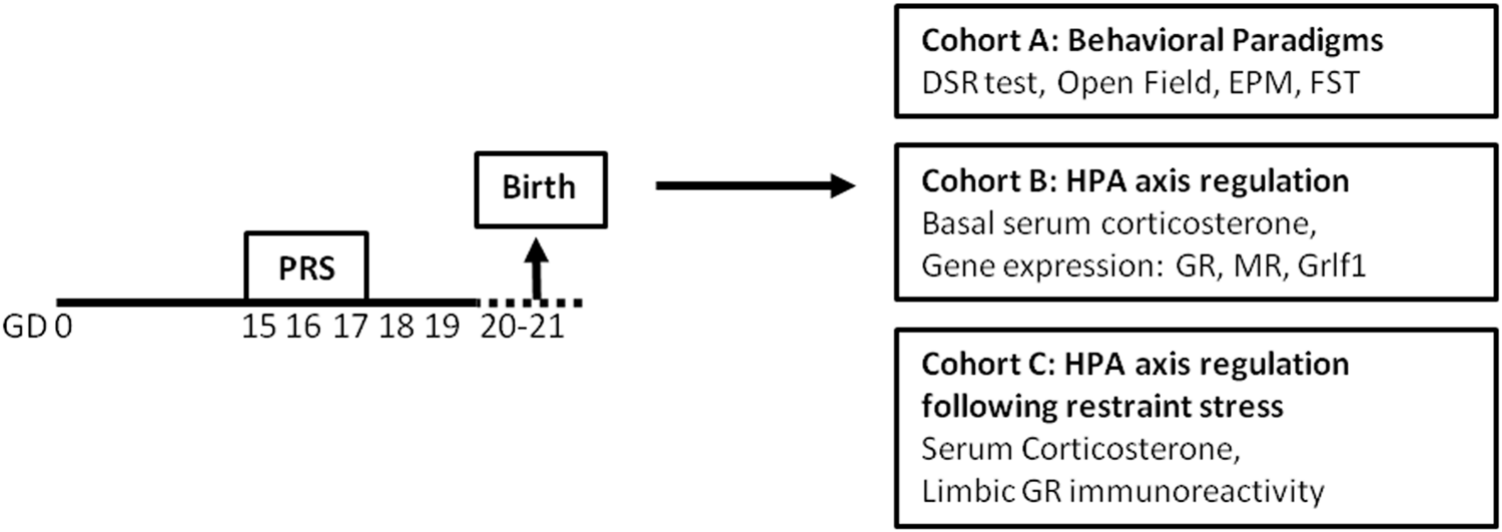
Study Design. Twelve week-old pregnant Dom and Sub females underwent 45 min of PRS on GDs 15–17 or remained undisturbed. After birth, litters were culled to equal size and randomly sorted into three cohorts: cohort A (n = 11–12 mice from PRS or undisturbed pregnancies from each strain) underwent behavioral testing, cohort B (n = 6) were sacrificed for assessment of basal HPA axis regulation, while cohort C (n = 10) underwent perfusion 24 hours following restraint stress to measure corticosterone levels and GR immunoreactivity in limbic areas involved in HPA axis regulation.

In order to determine the mechanisms underlying resilience and sensitivity to prenatal stress, the present study made use of selectively bred stress-resilient Dominant (Dom)^24,25^ and stress-sensitive Submissive (Sub) mice^26,27^. Dom and Sub mice were originally developed by selective breeding from the outbred Sabra line^28^ for their behavior in the Dominant-Submissive Relationship (DSR) social interaction paradigm^29^ in which constant pairs of mice compete for sweetened milk in successive test iterations, leading to the formation of robust and stable DSRs^29–32^. The repeated selective breeding of mice according to their performance in the DSR test for more than thirty generations yielded distinct mouse populations which develop strong and stable relationships of dominance and submissiveness^25,33^. Recently, prenatal restraint stress (PRS) wielded divergent consequences upon the postnatal development of Dom and Sub mice, subsequent to two distinct patterns of placental responses to prenatal adversity: placental GR recruitment in response to PRS enabled rapid postnatal weight catch-up after fetal growth retardation among Dom mice, while Sub mice remained underweight throughout adolescence^34^. Presently, we hypothesized that PRS would invoke adaptive or maladaptive prenatal programming of the stress response, respectively, among Dom and Sub mice. We further anticipated that such programming would be expressed in resilience or vulnerability to stress in adulthood, potentially mediated by limbic regulation of the HPA axis in response to adversity. Thus, in order to test the alterations in stress coping behavior incurred by PRS upon each mouse strain, dams underwent 45 min of PRS on gestational days (GD) 15, 16 and 17 (Fig. 1). Adult male offspring were then analyzed for depressive- and anxiety-like behavior, as well as their HPA axis regulation under both basal conditions and in response to acute stress.

## Results

### Cohort A: Prenatal Stress (PRS) exposure strengthened depressive- and anxiety-like behavior of Sub offspring

#### Sub offspring of Prenatal Stress (PRS) pregnancies demonstrate heightened depressive-like behavior in the Dominant-Submissive Relationship (DSR) test

**Figure 2:**
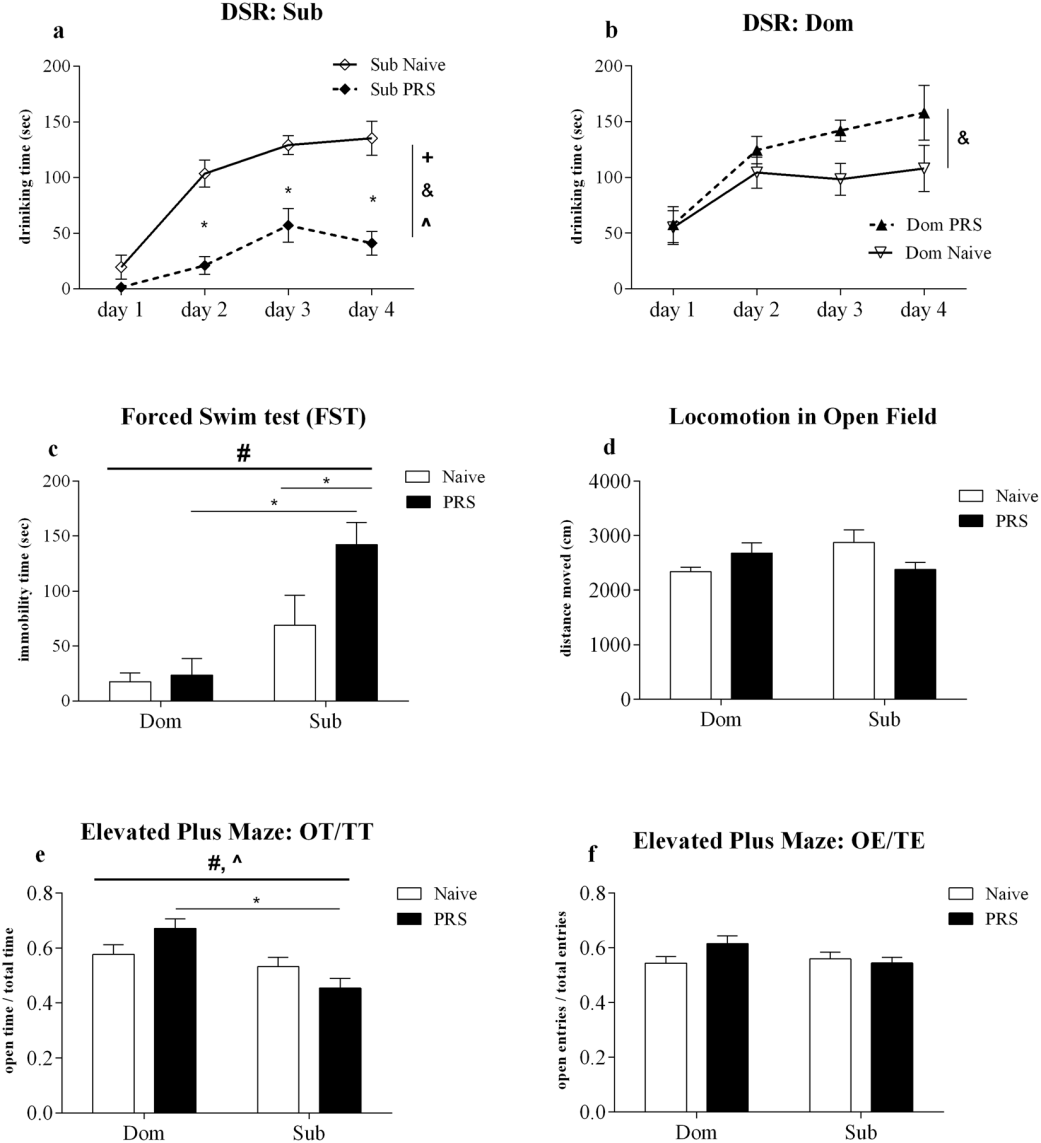
Prenatal Stress (PRS) exposure strengthened depressive- and anxiety-like behavior of Submissive (Sub) offspring. Sub PRS mice spent less time drinking sweetened milk when placed in the DSR test against their naïve counterparts (**a**), while Dom PRS mice display stable competition for food, in competition with Dom naïve (**b**). Sub PRS mice exhibited heightened immobility in the FST (**c**), relative to both Sub naïve and Dom PRS mice, without altered locomotion in the Open Field (**d**). In the EPM, open time: total time (OT/TT) indexes of Sub PRS mice were significantly lower than those of Dom PRS (**e**), without changes in the proportion of mice’s entries to open arms (OE/TE) (**f**). Data are presented as mean ± SEM, with independent variables contributing to statistical significance by two-way ANOVA indicated as: (#) strain effect; (+) PRS effect; (^) strain-PRS interaction. Between-measurement factor (time) indicated in (A,B) as (&). Bonferroni post-hoc pairwise comparisons indicated as *n = 11–12 (A,B); n = 6 (C); n = 10 (D,E,F).

Eight week old Sub offspring of PRS pregnancies showed subservience in the DSR test to their nonstressed counterparts (Fig. 2a, PRS effect: *F*_1,60_ = 39.69, *p* < 0.0001; time effect: *F*_3,60_ = 32.97, *p* < 0.0001; PRS-time interaction: *F*_3,60_ = 6.55, *p* < 0.001), significantly so on days 2(*t*_20_ = 5.30, *p* < 0.001), 3(*t*_20_ = 4.64, *p* < 0.001) and 4(*t*_20_ = 6.06, *p* < 0.001) according to Bonferroni post-hoc testing. No significant differences in drinking time were found between Dom mice born to PRS or undisturbed dams (Fig. 2b, PRS effect: *F*_1,60_ = 2.53, ns; time effect: *F*_3,60_ = 39.69, *p* < 0.0001; PRS-time interaction: *F*_3,60_ = 1.74, ns).

#### Sub offspring of PRS pregnancies demonstrated heightened depressive-like behavior in the Forced Swim Test (FST)

Sub mice spent far greater time immobile (Fig. 2c, main strain effect: *F*_1,20_ = 19.89, *p* < 0.001) in the FST, considered to be a marked acute stressor^35^. Bonferroni post-hoc testing found the effects of PRS upon immobility in the FST to be strain-dependent, reflected in a two-fold increase in immobility among PRS-Sub PRS mice relative to their naïve Sub counterparts (*t*_10_ = 2.73, *p* < 0.05) and six-fold relative to PRS-Dom (*t*_10_ = 4.41, *p* < 0.001), while PRS did not significantly affect immobility of Dom (*t*_10_ = 0.22, ns).

#### PRS exposure did not alter the general locomotor activity of Dom or Sub mice in the Open Field

In order to distinguish the above changes in depressive-like behavior from stimulatory effects, mice’s locomotion was measured in the Open Field (Fig. 2d). Two-way ANOVA detected no significant differences in cumulative distance as a function of strain (*F*_1,36_ = 0.50, ns) or PRS exposure (*F*_1,36_ = 0.20, ns).

#### Sub mice displayed anxiogenic effects of PRS in the Elevated Plus Maze (EPM)

While Dom mice spent a greater portion of time in the open arms of the EPM apparatus (Fig. 2e, OT/TT: time in open arms/total time, strain effect: *F*_1,36_ = 14.08, *p* < 0.001), relative to their Sub counterparts, PRS significantly strengthened this difference (strain-PRS interaction: *F*_1,36_ = 6.17, *p* < 0.05; among Naïve: *t*_18_ = 0.90, ns, PRS: *t*_18_ = 4.41, *p* < 0.001). No significant differences were found in the portion of mice’s entries to open arms (Fig. 2f, OE/TE: entries to open arms/total arm entries, strain effect: *F*_1,36_ = 1.22, ns; PRS effect: *F*_1,36_ = 1.30, ns).

### Cohort B: Inverse effects of PRS upon serum corticosterone levels and expression of HPA axis regulatory genes in the hippocampus of Dom and Sub mice

#### Sub offspring of PRS pregnancies demonstrated elevated serum corticosterone levels

**Figure 3:**
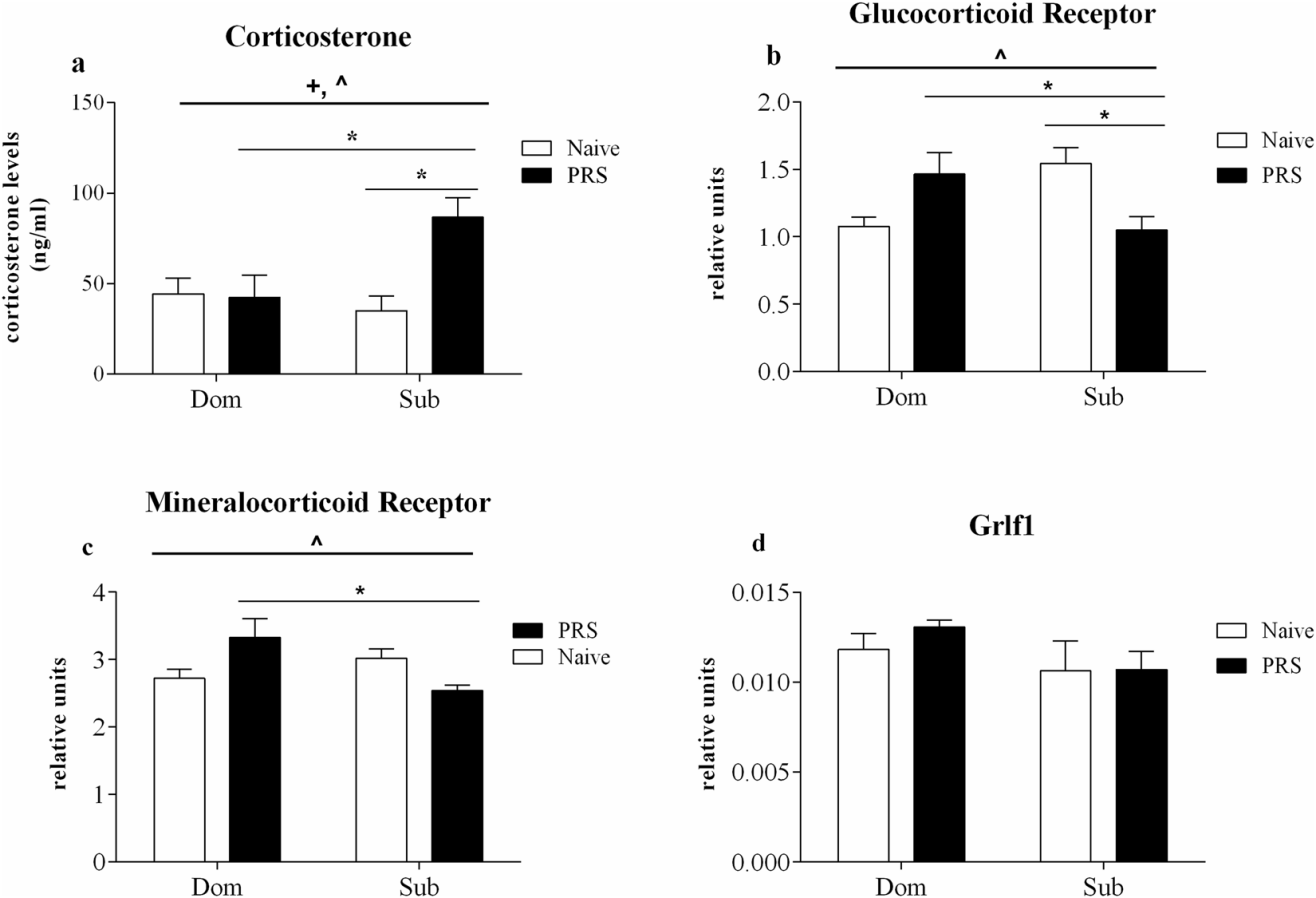
Prenatally stressed Submissive offspring develop long-term HPA axis dysregualtion. Sub offspring of PRS pregnancies demonstrated elevated basal serum corticosterone levels (**a**), alongside reduced hippocampal GR expression (**b**), while MR expression was reduced among Sub PRS offspring, relative to Dom counterparts (**c**). GR DNA-binding factor 1 (GRLF1) expression was unchanged by PRS (**d**). Data are presented as mean ± SEM, with independent variables contributing to statistical significance by two-way ANOVA indicated as: (#) strain effect; (+) PRS effect; (^) strain-PRS interaction. Bonferroni post-hoc pairwise comparisons indicated as *n = 5.

Two-way ANOVA detected elevated corticosterone levels among the offspring of PRS pregnancies (Fig. 3a, main PRS effect: *F*_1,16_ = 6.02, *p* < 0.05), due to the two-fold increase observed among Sub PRS mice only (strain-PRS interaction: *F*_1,16_ = 6.91, *p* < 0.05; Bonferroni pairwise comparison of PRS-Sub vs Naïve Sub: *t*_8_ = 3.60, *p* < 0.01, PRS-Sub vs PRS-Dom: *t*_8_ = 3.08, *p* < 0.05).

#### PRS elicits inverse effects upon hippocampal expression of HPA axis regulatory genes among Dom and Sub mice

Based upon its dual roles in mediation of negative feedback upon the stress-induced activation of the hypothalamic PVN^36^, as well as in regulation of mood and affect^37,38^, the hippocampus has been widely studied as a brain region wherein environmental stressors may achieve their detrimental effects in behavioral disorders among individuals sensitive to stress. Adaptive negative feedback upon the HPA axis following stress exposure is accepted to be largely dependent upon activity of the hippocampal Glucocorticoid Receptor (GR) and secondarily upon that of the Mineralocorticoid Receptor (MR)^4,5,39^. Presently, PRS yielded inverse effects among Dom and Sub mice born to PRS-exposed dams upon mRNA levels of both the GR, (Fig. 3b, PRS*strain interaction: *F*_1,16_ = 14.19, *p* < 0.01) and MR (Fig. 3c, *F*_1,16_ = 9.45, *p* < 0.01). Bonferroni post-hoc testing found PRS to significantly decrease GR expression in the hippocampus of Sub mice, when compared to Sub naïve (*t*_8_ = 2.98, *p* < 0.05) or to Dom PRS (*t*_8_ = 2.51, *p* < 0.05), while an apparently inverse effect of PRS upon Dom mice did not reach significance (*t*_8_ = 2.35, ns). PRS similarly influenced MR expression, which was elevated among Dom PRS, relative to Sub PRS (*t*_8_ = 2.72, *p* < 0.05), yet not significantly in comparison to Dom naïve (*t*_8_ = 2.44, ns) and without significant effects of PRS among Sub mice (*t*_8_ = 1.91, ns). In order to interrogate a potential mechanism mediating hippocampal GR expression, we measured the expression of the GR DNA-binding factor 1 (GRLF1, Fig. 3d), which enacts negative feedback upon GR transcription by blocking the NR3C1 gene promoter region when bound to activated GR in the nucleus^40,41^. PRS was not found to alter GRLF1 levels (*F*_1,16_ = 0.39, ns) nor were expression levels significantly different between strains (*F*_1,16_ = 2.89, ns).

### Cohort C: Dom and Sub mice exhibit opposing changes in serum corticosterone and hippocampal GR recruitment in response to restraint stress

In order to test the compound effects of PRS upon the ability of Dom and Sub offspring to cope with restraint stress as adults, three month-old mice born to either PRS or undisturbed pregnancies underwent restraint for 45 minutes. In order to measure the lasting effects of acute stress^42,43^ upon limbic sensitivity to glucocorticoids, mice were sacrificed 24 hours later, and HPA axis regulation was measured by serum corticosterone levels and GR immunoreactivity in the hippocampal CA1, basolateral amygdala (BLA) and the hypothalamal paraventricular nucleus (PVN).

#### Sub mice demonstrated markedly elevated serum corticosterone levels in response to restraint stress

**Figure 4:**
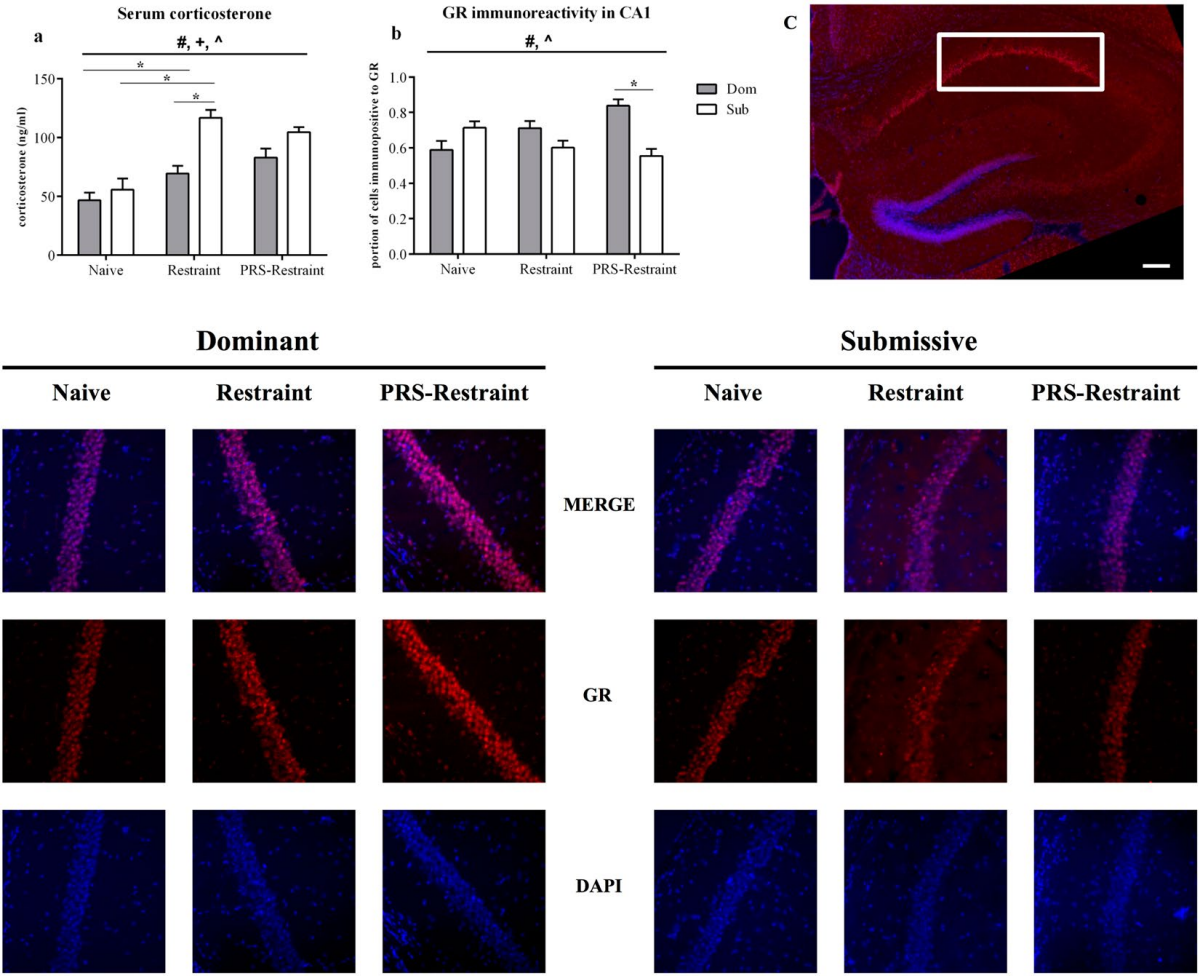
Serum corticosterone and glucocorticoid receptor (GR) immunoreactivity in the hippocampal CA1, 24 hours following restraint stress. Sub mice demonstrated markedly elevated serum corticosterone levels in response to restraint stress (**a**), while PRS-exposed Sub Dom mice effectively recruit hippocampal GR to the CA1 in response to restraint (**b**). Representative micrographs of the CA1 (white inset, **c**) from Naïve, Restraint and PRS-Restraint groups (lower pane). Scale bar 300 nm in c, 30 nm in lower pane. Blue channel: 4′,6-diamidino-2-phenylindole (DAPI); red channel: GR immunoreactivity visualized using Alexa 555 fluorophore. Data are presented as mean ± SEM, with independent variables contributing to statistical significance by two-way ANOVA indicated as: (#) strain effect; (+) restraint effect; (^) strain-restraint interaction. Bonferroni post-hoc pairwise comparisons indicated as *n = 10 (**a**), 24 images from 3 mice (**b**).

Serum corticosterone levels of mice born to PRS or undisturbed pregnancies, 24 hours following exposure to restraint stress, were higher among Sub mice than Dom (Fig. 4a, strain effect: *F*_1,54_ = 20.61, *p* < 0.001) and were elevated by the restraint procedure (stress effect: *F*_2,54_ = 24.37, *p* < 0.001), with a heightened reaction of Sub mice to stress (strain*stress interaction: *F*_2,54_ = 3.89, *p* < 0.05). Bonferroni comparisons between strains detected elevated serum corticosterone levels among restrained Sub mice, relative to Dom counterparts (*t*_18_ = 4.78, *p* < 0.001). No strain differences were evident among naïve mice (*t*_18_ = 0.91, ns) or those exposed to restraint following PRS (*t*_18_ = 2.18, ns). Comparison of restraint effects upon each mouse strain demonstrated significant influence among both Sub (*t*_18_ = 6.18, *p* < 0.001) and Dom mice (*t*_18_ = 2.31, *p* < 0.05), each in comparison to naïve. In neither strain was the influence of PRS detectable after restraint: (Bonferroni pairwise “restraint vs. restraint PRS”: Dom *t*_18_ = 1.37, ns; Sub *t*_18_ = 1.23, ns).

#### PRS-exposed Dom mice effectively recruit hippocampal GR to the CA1 region in response to restraint

The hippocampus is known to be a hub of the interplay between the HPA axis and regulation of mood and affect^37,38^. Given the pleiotropic role of GR in the CA1 region^44^, including modulation of pyramidal neuron excitability^45^, we measured GR immunoreactivity in the CA1 (Fig. 4c, white inset) of Sub and Dom mice born to PRS or undisturbed pregnancies, 24 hours following restraint. Alongside a significant strain effect (Fig. 4b, *F*_1,138_ = 7.16, *p* < 0.01), two-way ANOVA detected a highly significant, cross-over interaction between the effects of strain and stress upon GR levels (*F*_2,138_ = 12.75, *p* < 0.001). Post-hoc Bonferroni testing of PRS mice exposed to restraint found GR immunoreactivity to be far more pronounced among Dom than in Sub mice (*t*_46_ = 4.58, *p* < 0.001). At the same time, GR immunoreactivity in the BLA (Supplementary Fig. S3a) and the PVN (Supplementary Fig. S3b) was not found to be significantly different between strains (BLA: *F*_1,66_ = 0.84, ns; PVN: *F*_1,66_ = 0.03, ns), nor to be altered 24 hours following restraint (BLA: *F*_2,66_ = 2.05, ns; PVN: *F*_2,66_ = 2.93, ns).

## Discussion

In the current study, the adult offspring of Sub and Dom dams exposed to Prenatal Restraint Stress (PRS) demonstrated sharply contrasting abilities to cope with stress-inducing stimuli: while Sub mice born to PRS pregnancies developed depressive- and anxiety-like behavior, the ability of their Dom counterparts to contend with stressful challenges was not compromised by prenatal exposure to stress. Firstly, Sub offspring of PRS pregnancies showed subservience to their naïve counterparts in the DSR food-competition test (Fig. 2a), in contrast to Dom PRS mice (Fig. 2b). Thus, the inherited behavioral phenotypes of dominance and submissiveness of these selectively-bred mice^25,33^ were enhanced by PRS. The DSR paradigm was originally developed as a screening test for antidepressant properties of novel pharmaceutical interventions^29^, in which reduced drinking time is taken to reflect the decreased motivation and avoidance attributed to both submissive and depressive-like behaviors^25^. Noteworthy are recent works focusing upon individual differences in response to social stress^46,47^, which mark a qualified retreat from interpreting social defeat strictly as “depressive-like” behavior in rodents. With this taken into account, we nonetheless wish to suggest that the reduced drinking time of Sub PRS mice may partially model the induction of depression by prenatal stress reported among subpopulations of sensitive individuals^7^.

Following DSR testing, mice underwent the Forced Swim test (FST) to measure the degree to which mice assume passive stress-coping upon immersion^48^. Alongside the clear differences in the immobility time of naïve mice from each strain, PRS exposure further increased immobility among Sub offspring, without any apparent effect upon Dom mice (Fig. 2c). Previous reports of increased immobility in the FST among PRS-exposed offspring^49,50^ underline the distinct nature of the Dom mice’s resilience to depressive-like behavior following PRS. At the same time, mice’s locomotor activity in the Open Field (OF) remained constant (Fig. 2d), reinforcing the specific influence of PRS upon affective behavior in the DSR and FST. Next, given the common comorbidity of depression with anxiety disorders^51^, mice were tested for anxiety-like behavior in the Elevated Plus Maze (EPM), in which Sub PRS offspring displayed reduced exploratory behavior relative to their Dom counterparts (Fig. 2e), suggesting anxiogenic effects of PRS among Sub mice. While the anxiogenic effects of PRS upon Sub mice may have been anticipated based upon previous findings in rodents^52^, the resistance of Dom mice to the anxiogenic effects of PRS further highlights their resilience to the detrimental, transgenerational influence of PRS upon the offspring’s behavior.

Based upon the interplay between the hippocampal regulation of both the HPA axis and of mood and affect^6^, we measured the effects of PRS upon the expression of three key HPA axis regulatory elements in the hippocampus: the Glucocorticoid Receptor (GR), Mineralocorticoid Receptor (MR) and the Glucocorticoid receptor DNA-binding factor 1 (Grlf1)^40^. Among PRS offspring, Dom mice demonstrated markedly elevated hippocampal expression of both GR and MR, relative to their Sub counterparts (Fig. 3b,c). The GR and MR have been long studied for their interrelationships by which they facilitate the hormonal and behavioral responses to threatening stimuli, and subsequently enable return to homeostasis following the threat’s removal^6^. The partial attribution of Sub mice’s sensitivity to the depressive- and anxiogenic-like effects of PRS to their lower hippocampal GR and MR expression levels is in agreement with previous studies showing prenatal stress to lower hippocampal expression of both GR and MR among PRS offspring demonstrating altered stress response^3^.

In accordance with their reduced hippocampal expression of GR and MR, Sub PRS offspring demonstrated elevated basal serum corticosterone levels, relative to naïve controls (Fig. 3a), characteristic of detrimental prenatal programming of the HPA axis seen among vulnerable subpopulations^53^. In stark contrast, the elevated expression of both MR and GR (Fig. 3b,c) among Dom PRS mice appeared to enable more efficient negative feedback upon the HPA axis, as corticosterone levels of Dom PRS mice were not different from naïve (Fig. 3a), reminiscent of their resilience to stress in the behavioral paradigms (Fig. 2). Elsewhere, prenatal stress caused long-term elevation of offspring’s basal corticosterone levels^3^, as seen presently among Sub mice. Since early maternal care has been suggested to mediate the effects of prenatal stress upon the HPA axis regulation of offspring^54^, we measured dams’ latency to retrieve litters to the home cage nest. Dom and Sub dams showed similar retrieval times, while dams of neither strain showed effects of prenatal restraint stress (PRS) (Supplementary Fig. S2), suggesting that the differential influence of PRS upon offspring’s developing HPA axis was enacted *in utero*. At the same time, prenatal distress has been shown to alter postnatal maternal care^55^, which additional paradigms of maternal behavior, besides the Pup Retrieval test, may have captured. The current study, in which prenatally stressed dams raised their own offspring, was not designed to separate between the immediate (*in utero*) and long-term (postnatal) influence of PRS upon Dom and Sub offspring’s resilience or vulnerability to stressful challenge in adulthood. While offspring’s behavioral and biomolecular parameters reported above may be due in part to alterations in maternal care, the divergent placental responses of Dom and Sub mice to PRS^34^ suggest that the long-term behavioral and molecular consequences of prenatal adversity may also be attributed to *in utero* programming of the HPA axis^56,57^. The focus upon the intrauterine environment is supported by our recent findings demonstrating robust increase in placental enzyme 11beta-hydroxysteroid dehydrogenase 2, responsible for glucocorticoid metabolism, among Dom mice in response to prenatal stress^34^. Such findings parallel clinical reports in which prenatal maternal anxiety predicted long-lasting elevations in the offspring’s basal glucocorticoid levels^58^, alongside increased occurrence of psychopathology^59^ and altered stress response^60^.

Thus, in order to test involvement of the HPA axis in the trans-generational influence of PRS upon Dom and Sub mice’s response to acute stress, we measured serum corticosterone levels among both strains born to PRS or undisturbed pregnancies, one day following exposure to restraint stress. As anticipated, serum corticosterone levels were elevated among mice from both strains exposed to restraint, yet naïve to PRS (Fig. 4a). This response to restraint stress was far more pronounced among Sub mice, reaching levels twice those of their non-restrained counterparts. Serum corticosterone levels of restrained Sub mice were also considerably higher than their Dom counterparts, again pointing to PRS-induced dysregualtion of the HPA axis among Sub offspring. At the same time, the inter-strain differences in serum corticosterone levels were obscured among restrained mice born to PRS pregnancies, pointing to compound effects of this “double-hit”^61^ stress regimen, which were detectable even among largely resilient Dom mice. Furthermore, since the corticosterone levels of “PRS-Restraint” mice from each strain were not significantly different from those exposed to restraint alone, restraint stress appears to wield greater immediate influence upon corticosterone levels than does PRS exposure *in utero*.

Based upon the similar corticosterone levels of PRS-Restraint offspring from each strain, and given the central role which the GR plays in mediation of the behavioral and hormonal response to stress^62^, we speculated that the stark differences between Dom and Sub mice in the influence which PRS wields upon the offspring’s behavioral response to stress (Fig. 2) may be attributed to the sensitivity to glucocorticoids of brain regions regulating the stress response. Thus, we tested the impact of restraint upon GR immunoreactivity among mice of each strain born to PRS or undisturbed pregnancies in three critical points of limbic regulation of the HPA axis and affect: the hippocampal CA1, basolateral amygdala (BLA) and the hypothalamal paraventricular nucleus (PVN).

Hippocampal GR activation is considered essential to successful adaptation to stress, by both dampening the initial behavioral stress reaction and by promoting recovery through the elimination of stress-induced behavioral responses when they are no longer relevant^6^. Particularly, the CA1 is involved in the modulation of learned-helplessness behaviors^45^, such as the pronounced immobility demonstrated currently by Sub PRS mice in the FST (Fig. 2c). Analysis of hippocampal GR immunoreactivity in the CA1 24 hours following restraint yielded opposite effects among Dom and PRS Sub mice: GR immunoreactivity among Dom mice was markedly elevated, relative to their Sub counterparts (Fig. 4b). Mice were sacrificed 24 hours following restraint to allow full passage of the acute-phase effects of stress^42,43^. This time point was selected in order to measure the lasting effects of stress upon HPA axis regulation, suggested to indicate the transition from stress adaptation to psychopathologies^63^, since Dom and Sub mice show no differences in their corticosterone response to stress during the acute phase (Supplementary Fig. S1a,b). Thus, PRS appears to have enabled more effective recruitment of hippocampal GR among Dom mice in response to restraint stress, as demonstrated also in the gene expression levels of Dom PRS offspring under basal conditions (Fig. 3b).

The apparently adaptive response of GR expression among Dom PRS mice to stress may be at variance with previous reports showing prenatal stress to reduce GR expression among adult offspring, both in the CA1^3^ and in the entire hippocampus^64^. This aspect of prenatal programming of the developing HPA axis is considered a pathway by which prenatal stress exposure may lead to the later development of psychopathologies^38^, and mirrors the changes in GR expression currently found among Sub mice (Figs 3b, 4b). Thus, increased GR levels among Dom PRS offspring appeared in parallel with the remarkable resilience of these mice to the behavioral effects of PRS (Fig. 2), and may represent a potential mediator of their resistance to stress, as reported recently in mice subjected to social defeat^65^. Furthermore, the failure of Sub PRS mice to withstand acute stressful challenge in the FST (Fig. 2c) may be attributed in part to their reduced hippocampal GR expression (Figs 3b, 4b), as suggested elsewhere among chronically stressed rodents^66^.

**Figure 5:**
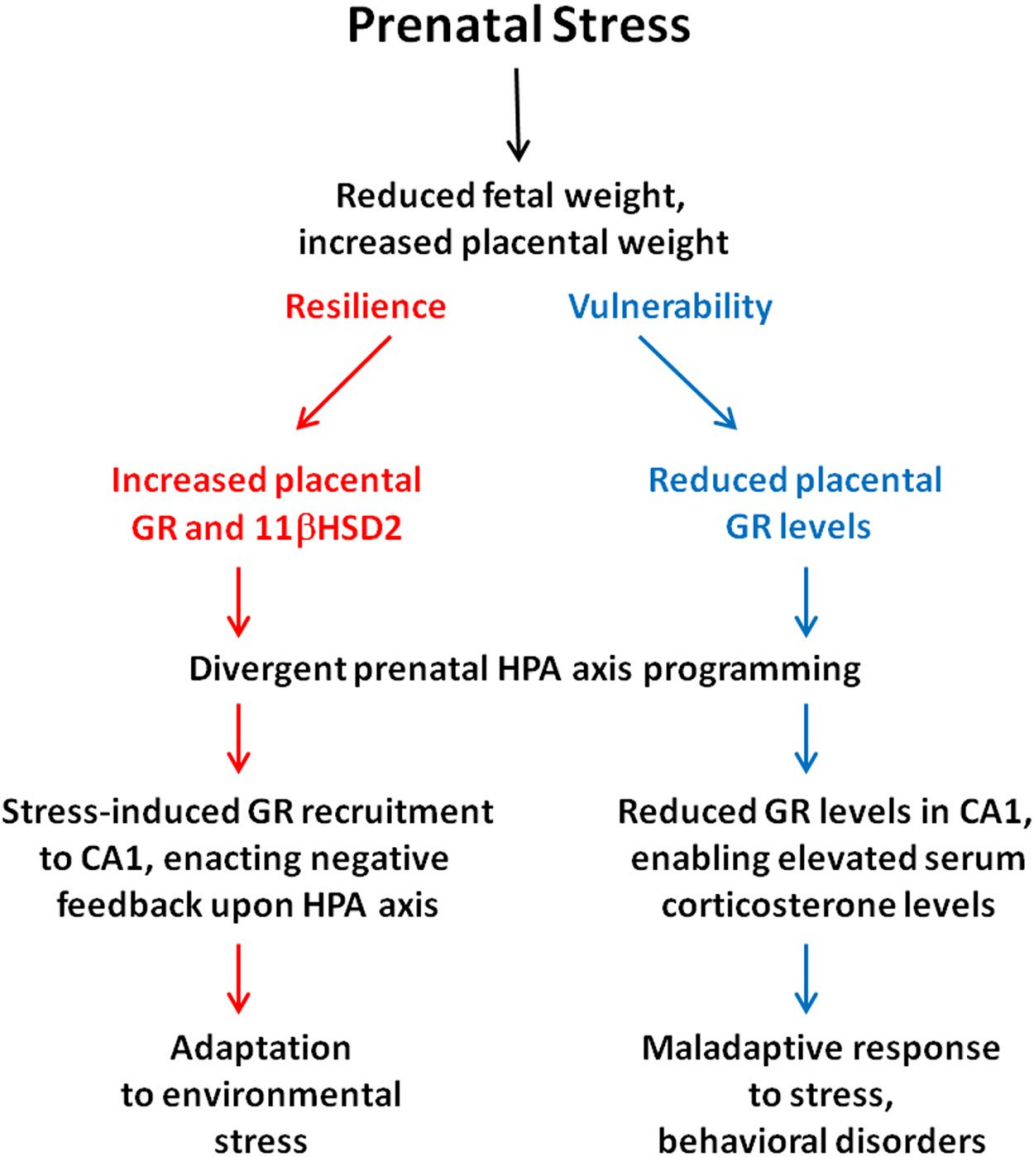
Proposed mechanism underpinning differential influence of prenatal stress upon offspring’s ability to cope with stress. Mild prenatal stress increases placental weight, at the expense of fetal weight, among both resilient and vulnerable offspring, yet placental levels of GR and 11βHSD2 are elevated only among resilient individuals. This augmentation of the placental enzymatic barrier between maternal glucocorticoids and the developing fetus enables adaptive prenatal programming of the HPA axis among resilient offspring. Upon stress exposure in adulthood, resilient individuals more effectively recruit GR to the hippocampus, facilitating negative feedback upon the HPA axis and enabling adaptive behavioral responses. glucocorticoid receptor (GR); 11beta-hydroxysteroid dehydrogenase 2 (HSD2); hypothalamic-pituitary-adrenal (HPA).

Activation of GR in the hippocampus by glucocorticoids leads to heightened glutamatergic innervation of GABA-releasing neurons of the bed nucleus of the stria terminalis (BNST), tonally strengthening the inhibition of CRF release from the PVN^36^. In parallel to the indirect inhibition enacted by hippocampal GR, the PVN itself possesses high levels of GR, which enacts direct inhibition of local CRF release^36^. While the early life environment has been shown to alter programming of hypothalamic gene expression^67^, we did not detect significant effects of either strain or restraint stress upon GR immunoreactivity in the PVN. These findings suggest that the wide-ranging roles of GR in the hypothalamus, including regulation of circadian rhythms and glucose metabolism, impart an evolutionary advantage to constant hypothalamic GR levels, regardless of behavioral phenotype or stress exposure. In contrast, the consistent levels of amygdalar GR immunoreactivity may appear surprising, given the BLA’s role in the processing of fear-related memory^68,69^, and the increased amygdalar GR levels reported among Major Depressive Disorder patients^70^. Furthermore, previous studies found prenatal elevation of maternal corticosterone levels to increase amygdalar GR expression among adult offspring^71^. However, the present study is the first to our knowledge to measure the compound effects of prenatal stress, followed by restraint among adult offspring, upon amygdalar GR immunoreactivity, and further study using a wider range of stressors may confirm this finding. Since the opposing influence of glucocorticoids in the amygdale and hippocampus are believed to complement each other, enabling short-term HPA axis activation, followed by longer-term recovery from stress^72^, the hippocampus-specific recruitment of GR among Dom mice may be partially responsible for their resilience to acute stress in the FST (Fig. 2c). Figure 5 outlines a proposed pathway by which the placental response to glucocorticoid exposure may facilitate adaptive prenatal programming of hippocampal GR expression among Dom mice and enable their resilience to stressful challenge in adulthood.

In the current study, PRS exposure markedly facilitated GR recruitment to the hippocampus in response to acute stress in adulthood, among Dom mice, which may be responsible for their resilience to stressful challenge. One limitation of the current work lies in the study of male offspring only. Male subjects were selected^73^ based on previous studies showing the long-term behavioral consequences of prenatal stress to be more robust among male rodents^56,62^, and to avoid potentially confounding effects of the adult female mouse’s estrous cycle upon behavioral parameters^56,74^. Future studies should be dedicated to understanding mechanisms mediating resilience and vulnerability to prenatal stress among female offspring. These findings nonetheless suggest proclivity to adaptive or maladaptive prenatal programming of the HPA axis in response to prenatal adversity to be inheritable traits, which in turn sharpens the active or passive coping styles of offspring to stress, as predicted in mice by their social dominance or submissiveness. Therefore, placental glucocorticoid signaling influences the HPA axis programming mechanisms by which the forthcoming generation’s adaptive or maladaptive style of coping with stress is determined, and that the starkly contrasting resilience or vulnerability of Dom and Sub mice respectively to PRS may be mediated by brain region-specific prenatal programming regulating hippocampal recruitment of the GR in response to stress.

## Materials and Methods

### Animals

Inbred Dominant (Dom) and Submissive (Sub) mice were selectively bred from the outbred Sabra line^28^ according to their behavior in the Dominant-Submissive Relationship (DSR) food competition test^29^, for over forty generations, yielding distinct mice populations which develop strong and stable dominant-submissive relationships^33^. Further behavioral study found Dom mice to display resilience to different stress-inducing stimuli^24,25^ while Sub mice demonstrated heightened sensitivity to stress^26,27^.

In order to test the transgenerational effects of prenatal restraint stress (PRS) upon Dom and Sub mice, three month old female mice were bred with males of their corresponding behavioral phenotype, and inspected twice daily for appearance of vaginal plug. The day on which plug appeared was considered gestational day (GD) 0, on which the males were removed. 18 pregnant dams from each strain were randomly selected to undergo 45 min of PRS on GD 15, 16 and 17 or to remain undisturbed throughout gestation. Sub mice were shown to develop long-term HPA axis dysregulation on GD19 following this PRS regimen^34^. After birth, litters (9 from each strain-treatment group: Dom-PRS, Dom-Naïve, Sub-PRS, Sub- Naive) were culled to equal size of 8 pups (4 male, 4 female) and otherwise left undisturbed. Male pups after weaning on PND 24 were rehoused (5/cage) on PND 24 with cagemates of the same strain-treatment group, randomly selected from the 9 litters comprising each group. Mice were subsequently resorted at the age of 8 weeks into three cohorts: Cohort A (n = 11–12 mice from PRS or undisturbed pregnancies from each strain) underwent the battery of behavioral testing described below, Cohort B (n = 5 mice from PRS or undisturbed pregnancies from each strain) were sacrificed by asphyxiation with CO2 and hippocampi extracted for qRT-PCR, as well as blood samples for serum corticosterone measurement, while Cohort C (n = 10 mice from PRS or undisturbed pregnancies from each strain) underwent perfusion 24 hours following 45 minutes of restraint stress in a 3 × 8 cm clear plastic restrainer with ventilation holes^75^, as well as serum corticosterone levels, alongside 10 naïve mice of each phenotype for assessment of corticosterone levels and GR immunoreactivity in limbic areas involved in HPA axis regulation. Figure 1 visualizes the study design and distribution of mice into cohorts.

With exceptions detailed in the DSR protocol below, mice were given standard laboratory chow and water *ad libitum*, and were housed in a colony room maintained at 22 ± 1 °C and illuminated at ∼200 lux on a 12 hour L:12 hour D cycle (lights on 07:00–19:00 hour)^76^. Mice were provided with ample nesting material for the creation of a thermoneutral environment^77^. The experiments were conducted in accordance with NIH/USDA guidelines, under the approval of the Ariel University Institutional Animal Care and Use Committee.

### Behavioral assessment

Mice of Cohort A underwent behavioral testing at the age of three months according the following regimen: Food was removed from cages 14 hours before initial DSR testing, after which food was reintroduced until the next 14-hour pre-testing period, for four consecutive days. After three days of rest and feeding *ad libitum*, general locomotion was assessed in the Open Field, immediately after which^78^, each mouse’s anxiety-like behavior was measured in the Elevated Plus Maze. Following a second three-day wash-out period, depressive-like behavior was measured in the Forced Swim test. All behavioral testing was conducted from 09:00–12:00 hour, the by experimenters blind to the mice’s group allocation.

#### Pup retrieval test

Maternal care was measured in a separate cohort of mice by the dam’s latency to retrieve pups, as previously described^79,80^, with the following modifications: on PND 4, 6 and 8, pups were separated for thirty minutes from the dam, after which three pups were returned to the dam’s cage at the opposite corner from the nest. The dam’s behavior during the test was video-recorded for off-line data analysis. Retrieval was scored when the dam picked up a pup in her mouth and transported it to the nest, with maximum retrieval time limited to 5 min.

#### Dominant-Submissive Relationship (DSR) test

The DSR food competition test was conducted in Sub-treatment vs Sub-naïve^37,81^ and Dom-treatment vs Dom-naïve^24^ setups, wherein “treatment” presently denotes prenatal restraint stress (PRS). Briefly, the Plexiglas apparatus consists of two identical chambers (8 × 8 × 12 cm) joined by a tunnel (3.5 × 3.5 × 25 cm) with a 0.5 cm diameter hole in the bottom center. A self-refilling feeder is connected to the tunnel, allowing a constant supply of sweetened milk. Eight week old male mice of each strain from different home cages were matched (offspring of PRS pregnancies vs. naïve) in the DSR test for four consecutive days. During each 16 h period preceding testing, mice were deprived of food (water was provided *ad libitum*), after which milk drinking times were recorded during each 5 minute DSR testing session.

#### The Forced Swim Test (FST)

As previously conducted^25,37^, mice were placed individually into a glass cylinder (30 cm in height, 10 cm in diameter) filled 25-cm high with water (25 ± 2 °C) for 6 min, during which time spent immobile was recorded by an observer blind to the treatment group’s identity. Mice were considered “immobile” if they displayed no additional activity other than that required to keep the head above water^82,83^. Following the FST, mice were dried with paper towels and warmed under a lamp for 10 min prior to return to their home cages.

#### Open Field

The Open Field (OF) test was used to control for potentially confounding influence of PRS upon mice’s general activity in the paradigms designed to measure their anxiety- and depressive-like behaviors. Light intensity was adjusted to ∼7 lux, to avoid light-induced hyperlocomotion artifact^84^ among stressed mice, as previously described^26^. The total distance travelled during 6 min in the 40 × 40 cm black plastic chamber was calculated using a digital camera operated by EthoVision 9.1 (Noldus, Netherlands).

#### Elevated Plus maze (EPM)

The Elevated Plus Maze (EPM) apparatus, comprised of two enclosed (10 × 45 × 40 cm) and two open (10 × 45 cm) arms extending from a common central platform (10 × 10 cm), was used to assess anxiety-like behavior^24,37^. Lighting was adjusted to ∼10 lux at the maze center, to optimize sensitivity to anxiogenic influence of stress, as performed previously^26^. The time each animal spent in the open and closed arms, and the frequency of entries to open and closed arms were recorded using a digital camera during each 5 minute session and calculated by EthoVision 9.1 (Noldus, Netherlands) software, with the location of mouse body center-point defining arm entry.

### Molecular, biochemical and immunohistochemical study

#### qRT-PCR analysis of hippocampal expression of HPA axis regulatory genes

Gene expression study by rt-PCR was performed as previously described^81,85^, with some modifications. Animals were euthanized in a CO_2_ chamber and hippocampi extracted immediately afterwards, frozen in liquid nitrogen, and stored at −80 °C. Total RNA was isolated using a 5′ Perfect Pure RNATissue kit, including a DNAse treatment procedure (5 Prime, Gentra, Valencia, CA, USA) and reverse transcribed to cDNA using a reverse transcription system (Promega, Madison, WI, USA). mRNA levels were then analyzed using SYBR Fast Universal Readymix Kit (KAPA, Woburn, MA, USA) in an MxPro3000 thermal cycler (Stratagene, Santa Clara, CA, USA). Primers were designed using the exon-exon junction principle and synthesized by Integrated DNA technologies (Coralville, IA, USA): Glucocorticoid receptor (GR): (F: 5′ TTCTGTTCATGGCGTGAGTACC 3′; R: 5′ CCCTTGGCACCTATTCCAGTT 3′); Mineralocorticoid receptor (MR): (F: 5′ ATGGAAACCACACGGTGACCT 3′; R: 5′ AGCCTCATCTCCACACACCAAG 3′); Glucocorticoid receptor DNA binding factor 1 (Grlf1): (F: 5′ CGAAGCCACAGGACTAAGCA 3′; R: 5′ TATGGTACCAGGGGGTCTGG 3′). Hypoxanthine Phosphoribosyltransferase (HPRT): (F: 5′ TGTTGTTGGATATGCCCTTG 3′; R: 5′ TTGCGCTCATCTTAGGCTTT 3′) served as an endogenous normalizer.

#### ELISA-based serum corticosterone assay

Mice were sacrificed between the hours of 9–12 am, the nadir of corticosterone levels among mice maintained in the current colony illumination conditions^86^. Peripheral circulation corticosterone levels were measured in serum samples prepared from trunk blood collected immediately after euthanasia and stored on ice for 1 hr before centrifuge at 3500 g for 7 min^24,37^, using a commercial ELISA kit detecting total serum corticosterone (MS E-5400 LDN, Nordhorn, Germany). Care was taken to ensure that the time from the mouse’s removal from the home cage until plasma collection did not exceed 2 minutes.

#### Immunohistochemical analysis of GR immunoreactivity in the hippocampal CA1, basolateral amygdala (BLA) and hypothalamic paraventricular nucleus (PVN)

Brain sectioning and immunostaining: Coronal sections (25 µm) were prepared from PFA-fixated brains (*n* = 3 for Naïve, Restraint and PRS-Restraint treated Sub and Dom mice) at −25 °C^87^, and stored in a cytoprotectant solution (30% Sucrose, 1% Polyvinyl-pyrrolidone (PVP40, Sigma-Aldrich, Rechovot, Israel) and 30% Ethylene glycol in PBS) at −20 °C for up to 4 weeks, enabling the staining of several groups in parallel. Immunohistochemical analysis was performed with the following modifications: brain sections from −0.8 (BLA and PVN) or −1.6 bregma (CA1) were re-fixated with 4% PFA for 10 min, permeabilized in Triton X-100 2.5% for 5 min and blocked with 5% goat serum and 0.3% Triton-X in PBS for 60 min at RT. Slices were then incubated with rabbit polyclonal anti-GR anibody (sc-1004, Santa Cruz Biotechnology, Santa Cruz, CA, USA), at concentration ratio of 1:200 in PBS with 1% goat serum overnight at 4 °C. Secondary hybridization was conducted for 1 hour at room temperature with an Alexa 555 anti-rabbit IgG antibody (Molecular Probes A-21137, Eugene, Oregon, USA) at 1:400. Sections were mounted in Fluoromount (Sigma-Aldrich F4680 Rehovot, Israel) with 2.5% DABCO anti-fading agent and 4′,6-diamidino-2-phenylindole (DAPI, Sigma-Aldrich D9542, Rehovot, Israel) at 0.5 µg/ml, coverslipped and sealed with acetone-based sealant.

Image capture and analysis: Analysis of fluorescence signal representing GR immunoreactivity was conducted using an upright fluorescent microscope (Zeiss LSM700, Carl Zeiss, Germany). Images were captured at x200 using a CCD camera (Axiocam MRm, Carl Zeiss, Germany) operated using Zen 2010 software (Carl Zeiss, Germany). For negative controls, omission of the primary antibody from the staining procedure was performed with no positive signal detected. Cells whose GR signal passed a constantly applied threshold were considered immunopositive for GR and the number of immunopositive cells within the ROI were counted, as well as the total cell count represented by DAPI-positive cellular nuclei, using ImageJ software (version 1.6, Wayne Rasband, National Institutes of Health, USA) after uniform background correction of each recorded image. The portion of cells in each ROI immunopositive for GR was calculated from the number of GR-positive cells/total cell number, within the identical field of view^88^. Analysis procedures were performed upon two adjacent images captured from each hippocampal CA1, in technical duplicate = 2 sections × 2 hippocampi × 2 images = 8 images per mouse, 24 images per experimental group. PVN images were captured immediately lateral of the third ventricle, and the BLA was located according to the morphology of the external capsule, such that each image included an entire BLA or PVN = 2 sections × 2 ROI × 1 image = 4 images per mouse, 12 images per group.

### Statistical analysis

Differences between mice’s behavior in DSR paradigm were analyzed by two-factor mixed-design ANOVA, with prenatal stress (PRS/Naïve) as between-measure independent variable and time (days) as within-subject factor. The remaining behavioral paradigms, as well as serum corticosterone and gene expression experiments were analyzed using two-way ANOVA, with prenatal stress (PRS/Naïve) and phenotype (Dom/Sub) as between-measure independent variables. Corticosterone levels and GR immunoreactivity of restrained offspring of PRS pregnancies were analyzed similarly, with stress modality (Naïve/Restraint/PRS-Restraint) and phenotype (Dom/Sub) as between-measure independent variables. Risk of Type I error due to multiple comparisons was mitigated by controlling familywise error rate (FWER) with Bonferroni post-hoc pairwise comparisons, for which *p* < 0.05 was considered the minimum for statistical significance. Analysis and mean ± SEM graph generation were performed using GraphPad Prism software (version 5.02).

### Data availability

The datasets analyzed in the current study are available in the Mendeley Data repository: 10.17632/x45ywkdxv6.1 (Fig. 2), 10.17632/7m56nw5zx4.1 (Fig. 3), 10.17632/6wzdgfnkxv.1, (Fig. 4), 10.17632/3w85zp3nzs.1 (Supplementary Fig. S1), 10.17632/55nmmk535t.1 (Supplementary Fig. S2), 10.17632/kwhwx9s7tv.1 (Supplementary Fig. S3) and 10.17632/ywgf8z3z6y.1 (Supplementary Fig. S4).

## Electronic supplementary material

Supplementary Figures
