# Supplementary material for: Social dominance predicts hippocampal glucocorticoid receptor recruitment and resilience to prenatal adversity
Source: Sci Rep. 2018 Jun 25;8:9595. doi: 10.1038/s41598-018-27988-9 (PMC6018627; doi:10.1038/s41598-018-27988-9)
Supplement: Supplementary file 1 — Supplementary Figures [file 41598_2018_27988_MOESM1_ESM.pdf]

Social dominance predicts hippocampal glucocorticoid receptor recruitment and resilience to prenatal adversity

Moshe Gross, Hava Romi, Ayala Miller and Albert Pinhasov*

Supplementary Figures

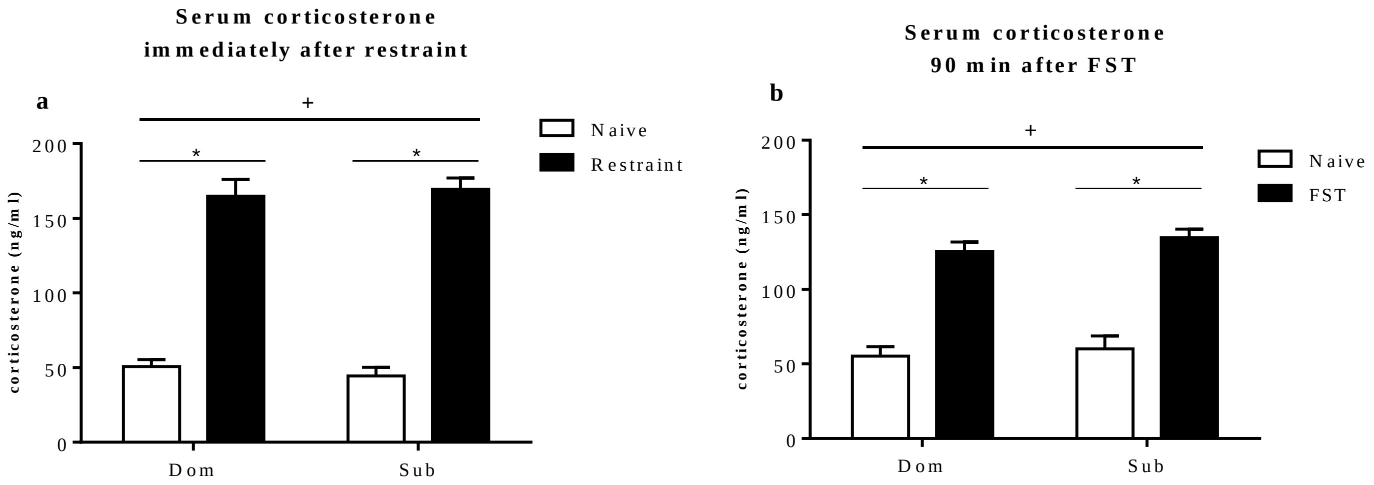

Supplementary Fig. S1: Dom and Sub mice demonstrate similar acute-phase corticosterone response to stress. Serum corticosterone levels of Dom and Sub mice sacrificed immediately following 45 minutes of restraint (**a**) or 90 minutes following the Forced Swim test (FST, **b**) demonstrate similar effects of stress (**a**: $F_{1,16}$ = 244.8, p<0.001; **b**: $F_{1,16}$ = 110.0, p<0.001), without between-strain differences (**a**: $F_{1,16}$ = 0.01, ns; **b**: $F_{1,16}$ = 1.03, ns). Data are presented as mean ±SEM, with independent variables contributing to statistical significance by two-way ANOVA indicated as: (+)stress effect. Bonferroni post-hoc pairwise comparisons indicated as *(p<0.001); n=5.

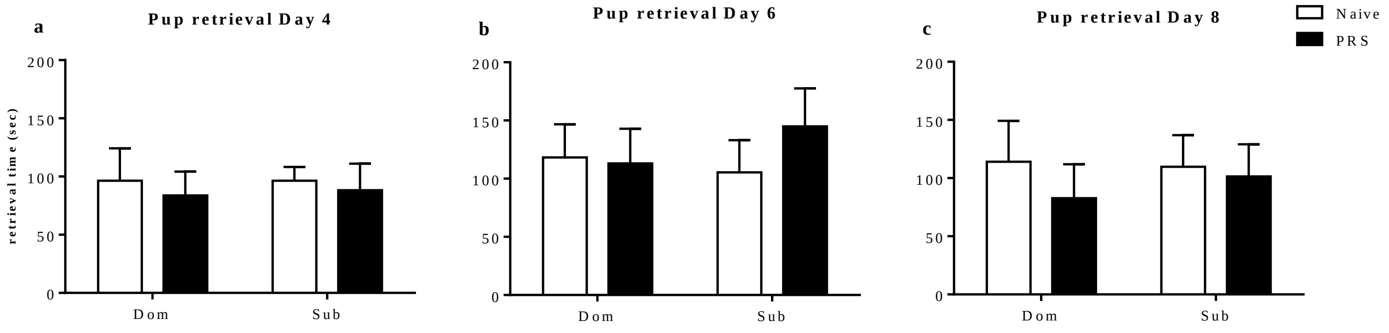

Supplementary Fig. S2: Pup Retrieval test of naïve and prenatally-stressed Dom and Sub mice. Dom and Sub dams showed similar latency to retrieve pups to the home cage nest on Day 4 (**a**, $F_{1,56} = 0.05$, ns), Day 6 (**b**, $F_{1,56} = 0.10$, ns) and on Day 8 (**c**, $F_{1,56} = 0.06$, ns). Dams of neither strain showed effects of prenatal restraint stress (PRS) upon retrieval latency (Day 4: $F_{1,56} = 0.10$, ns; Day 6: $F_{1,56} = 0.33$, ns; Day 8: $F_{1,56} = 0.43$, ns). Data are presented as mean ±SEM; n=15 dams.

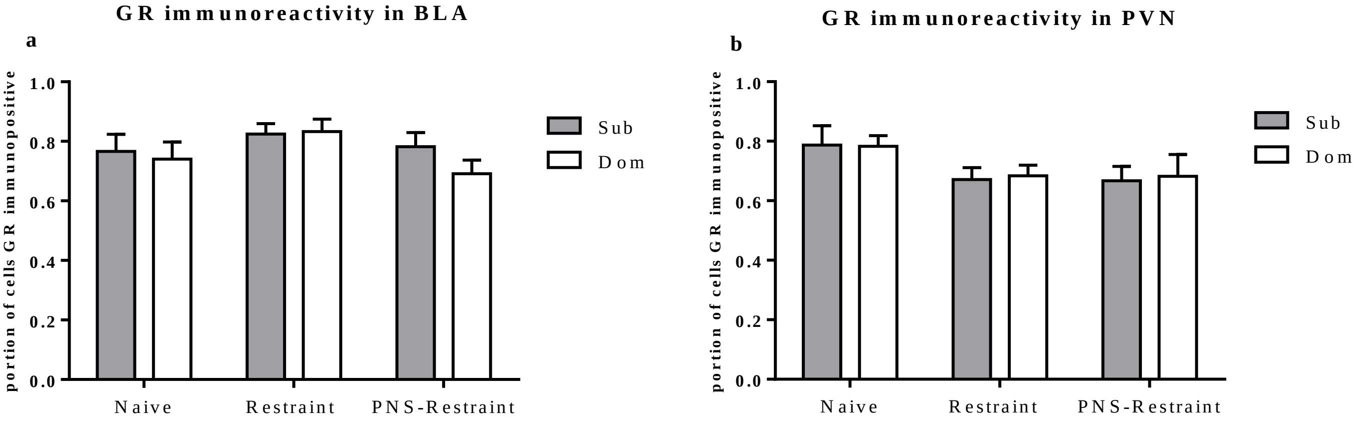

Supplementary Fig. S3: Glucocorticoid receptor (GR) immunoreactivity in basolateral amygdala (BLA), and hypothalamal paraventricular nucleus (PVN) of Dom and Sub mice, 24 hours following restraint stress. GR immunoreactivity in the BLA (**a**) and the PVN (**b**) was not found to be significantly different between strains (BLA: $F_{1,66} = 0.84$, ns; PVN: $F_{1,66} = 0.03$, ns), nor to be altered 24 hours following restraint (BLA: $F_{2,66} = 2.05$, ns; PVN: $F_{2,66} = 2.93$, ns). Data are presented as mean ±SEM, n=12 images from 3 mice.

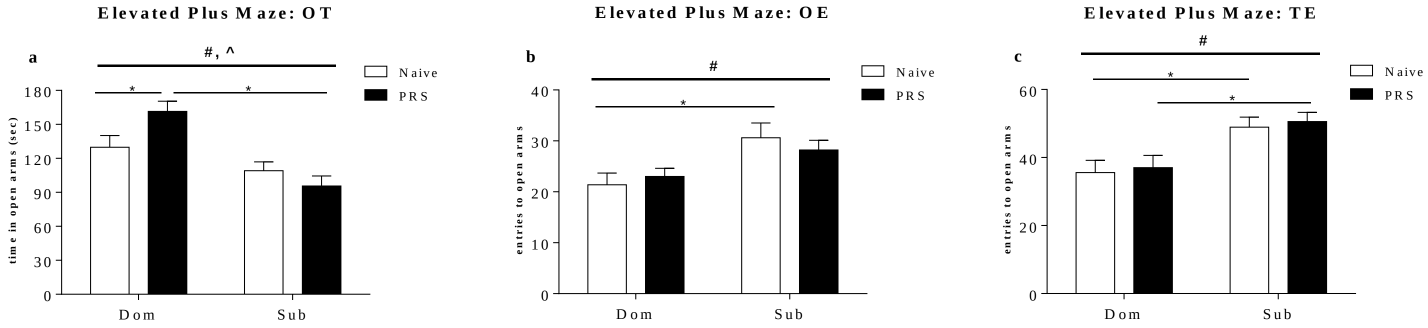

Supplementary Fig. S4: Open arm dwell times and arm entry frequencies of Dom and Sub mice in the Elevated Plus Maze (EPM). Dom mice spent more time in the open arms of the EPM (**a**, strain effect: $F_{1,36} = 22.91$, $p<0.001$), a tendency which prenatal restraint stress (PRS) strengthened (strain-PRS interaction: $F_{1,36} = 6.23$, $p<0.05$). Sub mice displayed heightened locomotion in the EPM, as indicated by more frequent open arm (**b**, strain effect: $F_{1,36} = 10.35$, $p<0.01$) and total arm entries (**c**, strain effect: $F_{1,36} = 17.29$, $p<0.001$). Data are presented as mean ±SEM; n=10 mice.
